# Sortilin knock-down alters the expression and distribution of cathepsin D and prosaposin and up-regulates the cation-dependent mannose-6-phosphate receptor in rat epididymal cells

**DOI:** 10.1038/s41598-023-29157-z

**Published:** 2023-03-01

**Authors:** Andrea Carolina Aguilera, Natalia Leiva, Pablo Ariel Alvarez, Georgina Pulcini, Laura Lucía Pereyra, Carlos Ramón Morales, Miguel Ángel Sosa, Lorena Carvelli

**Affiliations:** 1 CONICET, Facultad de Ciencias Médicas, Universidad Nacional de Cuyo, M5500 Mendoza, Argentina; 2 Facultad de Ciencias Exactas y Naturales, Universidad Nacional de Cuyo, M5500 Mendoza, Argentina; 3 IHEM-CONICET, Facultad de Ciencias Médicas, Universidad Nacional de Cuyo, M5500 Mendoza, Argentina; 4 Faculty of Medicine, McGill University, Montreal, QC H3A0C7 Canada

**Keywords:** Lysosomes, Golgi

## Abstract

The selective transport to lysosomes can be mediated by either mannose-6-phosphate receptors (CD-MPR and CI-MPR) or sortilin. In mammalian epididymis, some lysosomal proteins are secreted into the lumen through unknown mechanisms. To investigate the underlying mechanisms of lysosomal protein transport in epididymal cells we studied the expression and distribution of cathepsin D (CatD) and prosaposin (PSAP) in a sortilin knocked down RCE-1 epididymal cell line (RCE-1 KD) in comparison with non-transfected RCE-1 cells. In RCE-1 cells, CatD was found in the perinuclear zone and co-localize with sortilin, whereas in RCE-1 KD cells, the expression, distribution and processing of the enzyme were altered. In turn, PSAP accumulated intracellularly upon sortilin knock-down and redistributed from LAMP-1-positive compartment to a perinuclear location, remaining co-localized with CatD. Interestingly, the sortilin knock-down induced CD-MPR overexpression and a redistribution of the receptor from the perinuclear zone to a dispersed cytoplasmic location, accompanied by an increased co-localization with CatD. The increase in CD-MPR could result from a compensatory response for the proper delivery of CatD to lysosomes in epididymal cells. The intracellular pathway taken by lysosomal proteins could be an approach for addressing further studies to understand the mechanism of exocytosis and therefore the role of these proteins in the epididymis.

## Introduction

The mammalian epididymis plays a crucial role in the development of the sperm fertilizing capacity, providing a proper environment for gamete maturation^1^. The composition of the epididymal luminal fluid varies among mammalian species and organ regions (caput, corpus and cauda)^2,3^. Caput lining cells secrete 70–80% of the total epididymal proteins^4,5^, which play a critical role in sperm maturation. Among these proteins, there are several enzymes that participate in the remodeling of the sperm surface as a stage in the maturation of gametes^6–8^. Some of these enzymes are lysosomal proteins, which are synthesized by the epididymal epithelium and secreted into the organ lumen^9,10^. The secretion mechanism of these enzymes is still poorly understood, and their participation in sperm maturation is not fully known.

The lysosomal protease cathepsin D (CatD) has been proposed as a key enzyme in the remodeling of the sperm plasma membrane and improvement of its fertilizing capacity^11–13^. Other authors have suggested the participation of CatD in the processing of other lysosomal proteins such as prosaposin (PSAP)^14^. Secreted CatD and PSAP have been shown to be acquired by the gametes in the epididymal duct, suggesting a role of these proteins in sperm maturation^11–13^. As in other organs of the male reproductive tract, the epididymis secretory activity has been found to be hormone dependent. Previous reports have shown that androgen deprivation induces changes in the expression, intracellular distribution, and secretion, of both CatD and PSAP in rat epididymis^9,10,15,16^. However, the mechanisms that regulate the secretion of these proteins are not fully understood.

In most eukaryotic cells, lysosomal enzymes are selectively transported to the lysosomes via specific membrane mannose-6-phosphate receptors (MPRs). The MPRs recognize mannose-6-phosphate (M6P)-bearing enzymes to target them selectively from the trans-Golgi network (TGN) to the lysosomes^17–21^. Two forms of MPRs have been described so far; the cation-dependent (CD-MPR) and the cation-independent (CI-MPR)^17–22^, which coexist in most eukaryotic cells. Both types of MPRs are mostly located in endosomes, TGN, and the plasma membrane^23^. In some cell types, the CD-MPR present in the plasma membrane has been proposed to play a role in the exocytosis of M6P-bearing ligands^24–26^. Particularly, in breast cancer cells, the transport and secretion of CatD is driven by a CD-MPR delivery route, which is regulated by estrogen^27^. Although CatD is widely known to be a ligand for MPRs, other alternative routes have been described for this enzyme^28^. Experimental evidence indicates that CatD can also be transported by the membrane receptor sortilin, through its interaction with PSAP, the natural ligand of this receptor^29–31^. Studying the role of sortilin in the intracellular transport of PSAP-CatD complexes^29^ might be key to elucidate whether this receptor participates in the CatD secretion by the epididymal cells^16^.

Sortilin is a member of the vacuolar protein sorting 10 protein family (Vps10p) expressed in several mammalian cell types as well such as neurons, hepatocytes, and macrophages^32^. Many authors suggest that an altered expression or function of sortilin leads to the development of several diseases^33^. The highest density of sortilin is found in the TGN, where it is involved in the trafficking of multiple ligands to either endosome/lysosome compartments or to the secretory pathway. One tenth of this receptor is present at the plasma membrane, playing a role in either the internalization of ligands or the intracellular signaling^33^. Sortilin is involved in the trafficking of lysosomal proteins such as, acid sphingomyelinase, GM2AP, PSAP, cathepsin H and less frequently, CatD^30^. In mice, the sortilin gene inactivation did not result in any perceptible lysosomal pathology and the endogenous and exogenous PSAP continued to be targeted to lysosomes, suggesting the existence of an alternative receptor complementing the sorting role of sortilin^34^.

The purpose of this study is to understand the mechanism involved in the transport of lysosomal proteins in the epididymis and therefore to infer its role in the conditioning of the proper luminal environment for sperm maturation. To this end, epididymal cells from rat caput (RCE-1)^35^ were knocked down for sortilin to evaluate the expression and distribution of the lysosomal proteins CatD and PSAP, as well as CD-MPR. Our results suggest that the transport of lysosomal proteins by CD-MPR and sortilin in rat epididymal cells is accomplished through a concerted mechanism of both receptors and provide new insights to understand the processes associated with sperm maturation carried out in the epididymis.

## Materials and methods

### Reagents and antibodies

The rabbit anti-CD-MPR antiserum was gently provided by Dr. Annette Hille-Rehfeld (Georg-August Universität, Göttingen, Germany). The goat anti-CatD antiserum was purchased from Santa Cruz Biotechnology (sc-6487 Dallas, TX, USA). The polyclonal rabbit anti-PSAP antiserum was produced by Dr. Carlos R. Morales (McGill University, Montreal, Canada). The mouse anti-sortilin antibody was purchased from BD Biosciences (cat. 612100, New Jersey, US). The mouse anti-LAMP-1 antiserum was from Invitrogen (MA1-164, Burlington, ON, Canada). The anti-rabbit conjugated to Alexa 488 (Cat # A-21206), anti-mouse conjugated to Alexa 488 (Cat # A-21202), anti-mouse conjugated to Alexa 594 (Cat # A-21203) and anti-goat conjugated to Alexa 594 (Cat # A-11058) antibodies were from Invitrogen (Burlington, ON, Canada). The HRP-conjugated anti-goat IgG antiserum was obtained from Merck Millipore, (cat.401515, Calbiochem ® Burlington, MA, USA), the HRP-conjugated anti-rabbit IgG was purchased from Sigma (A9169, St. Louis, MO, US) and the HRP-conjugated anti-mouse IgG (whole molecule) was purchased from Sigma (A9044, St. Louis, MO, US). Nitrocellulose (NC) membranes (0.45 μm pore size) were purchased from GE Healthcare (Cat# 10600003, Germany). The chemiluminescent reagent was prepared with 1.25 mM luminol (Cat# A8511, Sigma-Aldrich, St Louis, MO, US), and 198 μM *p-*coumaric acid (Cat #722812, Sigma-Aldrich, St Louis, MO, US) in 100 mM Tris–HCl (pH 8.5)^36^. The transfection reagent used was PolyFect ® Transfection Reagent (Cat# 301105, Qiagen, Mississauga, ON, Canada). All other chemicals and reagents were purchased from Sigma-Aldrich (St. Louis MO, USA) unless otherwise specified.

### Cell culture

The caput epididymal cell line RCE-1 was gently provided by Dr. Daniel G. Cyr (Université du Québec, Laval, Canada)^35^. Cells were cultured in plates coated with extracellular matrix (EM, Cat# E1270, Sigma-Aldrich, St Louis, MO, US) diluted 1:10 in Dulbecco’s modified Eagle medium (DMEM)/Ham nutrient mixture F12 supplemented with 2 mM l-glutamine, 10 μg/mL insulin, 10 μg/mL transferrin, 80 ng/mL hydrocortisone, 10 ng/mL epidermal growth factor, 10 ng/mL cAMP, 50 IU/50 μg/mL penicillin–streptomycin (Gibco, MA, US) and with 10% fetal bovine serum (Gibco, MA, US) at 33 °C in a humidified chamber with 5% CO_2_.

### Transfection

The DNA plasmid pSilencer™ 3.1-H1 neo (Ambion, Austin, TX) that contained a subcloned sortilin siRNA template oligonucleotide (AAGGTGGTGTTAACAGCAGAG) was used in this study^37^. Cells were transfected following the manufacturer’s instructions. Briefly, DNA and Polyfect transfection reagent were pre-incubated for 10 min at room temperature to allow complex formation. Afterwards, cells were washed once with sterile phosphate-buffered saline (PBS) and supplemented DMEM culture medium was added with the transfection reagents and incubated for 24–48 h. The transfected clones (RCE-1 KD cells) were selected with 500 µg/mL geneticin (Cat # 11811031, Gibco, MA, US). Cells were mock-transfected with an empty pSilencer TM 3.1-H1 neo-vector as control (RCE-1 M cells)^30^.

### Immunoblotting

Cells were lysed and homogenized with lysis buffer (PBS containing 1% Nonidet P-40 and 1 mM PMSF) followed by sonication. Proteins (40 μg) from homogenates were resuspended in Laemmli's buffer^38^, boiled for 5 min and subjected to 10% SDS-PAGE. The inmunoblotting procedure was carried out according to Aguilera et al.,^39^. Membranes were incubated overnight at 4 °C with either; anti-CatD (1:1000), anti-PSAP (1:300), anti-sortilin (1:1000), or anti-CD-MPR (1:250) antisera, all diluted with 1% BSA (bovine serum albumin) in PBS-T (PBS containing 0,1% Tween 20). After that, membranes were washed three times with PBS-T and incubated with the corresponding HRP-conjugated secondary antibody for 2 h. Specific bands were detected by enhanced chemiluminescence and quantified by densitometric scanning using an ImageQuant LAS 4000 Luminescent Image Analyzer (Fujifilm Life Sciences, NY, US). Band intensities were quantified by densitometry using the ImageJ software (Image Processing and Analysis in Java; National Institutes of Health, Bethesda, MD, USA). As loading control, nitrocellulose membranes were stained with Ponceau S (Cat# P3504, Sigma-Aldrich, St Louis, MO, US). From the Ponceau S staining three bands were taken as references and quantified individually by densitometry with ImageJ. Then, the bands of each protein under study (Sortilin, CD-MPR, PSAP or CatD) were normalized against the mean of the ponceau selected bands.

### Indirect immunofluorescence and confocal microscopy

For IFI studies, cells were cultured on 12 mm glass coverslips under the experimental conditions described above. When a 50% confluence was reached, cells were washed once with PBS and fixed with 2% paraformaldehyde for 20 min. Afterwards, cells were permeabilized with 0.1% saponin for 15 min, washed three times with PBS and blocked with 5% horse serum for 30 min. Cells were then incubated overnight at 4 °C with either; anti-CatD (1:200), anti-PSAP (1:1000), anti-sortilin (1:50), anti-CD-MPR (1:250) or anti-LAMP-1 (1:200) antibody, all diluted in PBS containing 5% horse serum (PBS-HS). Cells were washed three times with PBS and incubated with the corresponding fluorochrome-conjugated secondary antibodies diluted in PBS-HS for 90 min. Cell nuclei were stained with 0.007 mg/mL Hoeschst 33342, and coverslips were mounted using Mowiol 4–88 mounting solution. The immunofluorescent staining was analyzed under an Olympus FV 1000 confocal microscope and images were acquired using the FV 10-ASW 1.7 software (Olympus, Japan). The images acquisition was set at 20 ms/Pixel (sampling speed) and 3 times integration count (Kalman = 3).

### Image and quantitative co-localization analysis

The image analysis and quantification were carried out using ImageJ software from 40 to 70 cells for each cell line. The co-localization degree of proteins was analysed by the Manders correlation coefficient (MCC)^27^. MCC-M1 and MCC-M2 were used to determine the amount of each protein that co-localized with other protein in the cells. The MCC values ranged from 0 to 1, indicating no co-localization or complete co-localization, respectively. Proteins were considered to co-localize when the values of the coefficients were above 0.5. The analysis was done using the JACoP plugin (Just Another Co-localization Plugin; NIH)^40^ of the ImageJ software.

### Statistics

Data were analysed by the Tukey–Kramer multiple comparisons test. The level of significance was set at p < 0.05. At least three independent experiments were performed in each case.

## Results

### Sortilin is expressed in epididymal cells and co-localizes with cathepsin D

**Figure 1:**
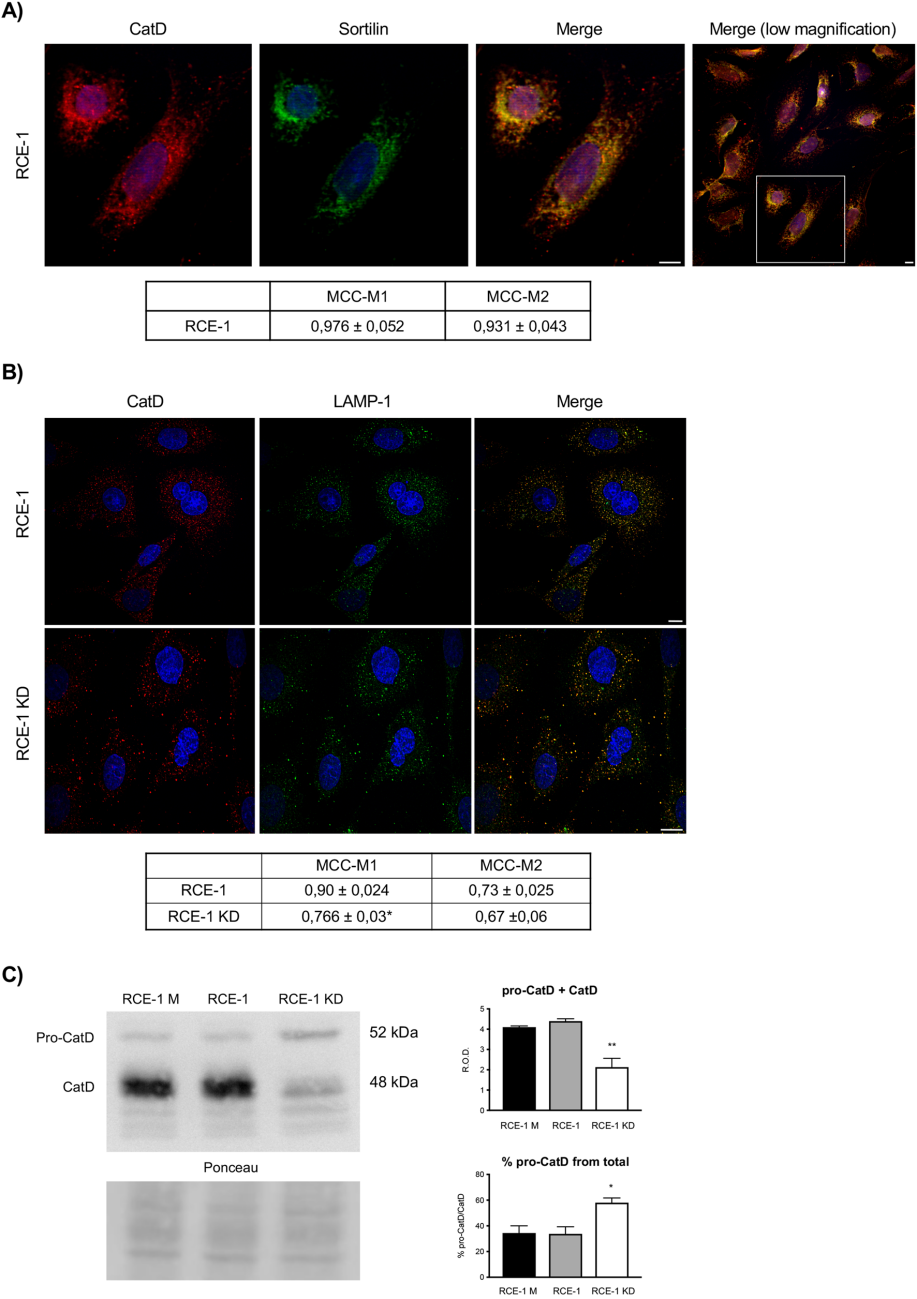
Effect of sortilin depletion on the distribution and processing of Cathepsin D in RCE-1 cells. (**A**) Immunofluorescence staining of sortilin and CatD in the RCE1 cells. Confocal representative images and quantification of co-localization of sortilin and CatD (MCC-M1); CatD and sortilin (MCC-M2). The shown cells were taken from the framed area of the lower magnification image (right merge). (**B**) Representative immunofluorescence staining of CatD and LAMP-1 in RCE1 and RCE-1 KD cells. Quantification of co-localization of LAMP-1 and CatD (MCC-M1); CatD and LAMP-1 (MCC-M2). Values are expressed as the means of Manders colocalization coefficients 1 and 2 (MCC-M1 and MCC-M2, respectively) ± SEM. (*) significant difference from RCE-1 (p < 0.01). Scale bars = 10 μm. (**C**) Representative immunoblot of CatD in RCE-1 M (mock-depleted), RCE-1 and RCE-1 KD cells. Bands intensities of pro-CatD (52 kDa) and CatD (48 kDa) were quantified separately. Bars represent the means of the total CatD (pro-CatD + CatD), as relative optical densities (R.O.D.) ± SEM (upper histogram) and the means of pro-CatD percentage in each sample ± SEM (lower histogram) from four independent experiments. (*) and (**) significant differences (p < 0.01 and p < 0.05, respectively). Ponceau S staining were used as loading control. The full-lenght immunoblot is presented in Supplementary Fig. 2.

Sortilin was found to be highly expressed in the epididymal RCE-1 cell line, displaying a perinuclear and cytoplasmic punctate location (Fig. 1A). In addition, a high co-localization of sortilin with the lysosomal protease CatD was observed (Fig. 1A). These results suggest that sortilin could participate in sorting and delivery of CatD to lysosomes in principal epididymal cells.

Based on these findings, it was of interest to determine if the sortilin knock-down could impact on the expression and distribution of the lysosomal proteins CatD and PSAP. For this purpose, we have established a stable sortilin-silencing RCE-1 epididymal cell line (RCE-1 KD) by transfection with the pSilencer™ sortilin siRNA plasmid. The sortilin depletion was confirmed by immunoblotting and immunofluorescence staining in selected transfected clones (Supplementary Fig. 1A,B). With this silencing method, a decrease of about 85.2 ± 0.2% in the expression of sortilin was attained.

### Sortilin knock-down (KD) alters distribution and processing of cathepsin D

The sortilin knock-down induced a decrease of intracellular CatD (Fig. 1C) and a slight redistribution of CatD from the perinuclear to a cytoplasmic dispersed location (Fig. 1B). In turn, CatD displayed lower co-localization with the lysosomal protein LAMP-1 (Fig. 1B), indicating that the enzyme could be detoured from the route to lysosomes. This finding was consistent with the decreased cleavage of pro-CatD (52 kDa) to a processed form of 48 kDa, as observed by immunoblotting (Fig. 1C).

### The sortilin knock-down (KD) alters the expression and intracellular distribution of prosaposin in RCE-1 cells

**Figure 2:**
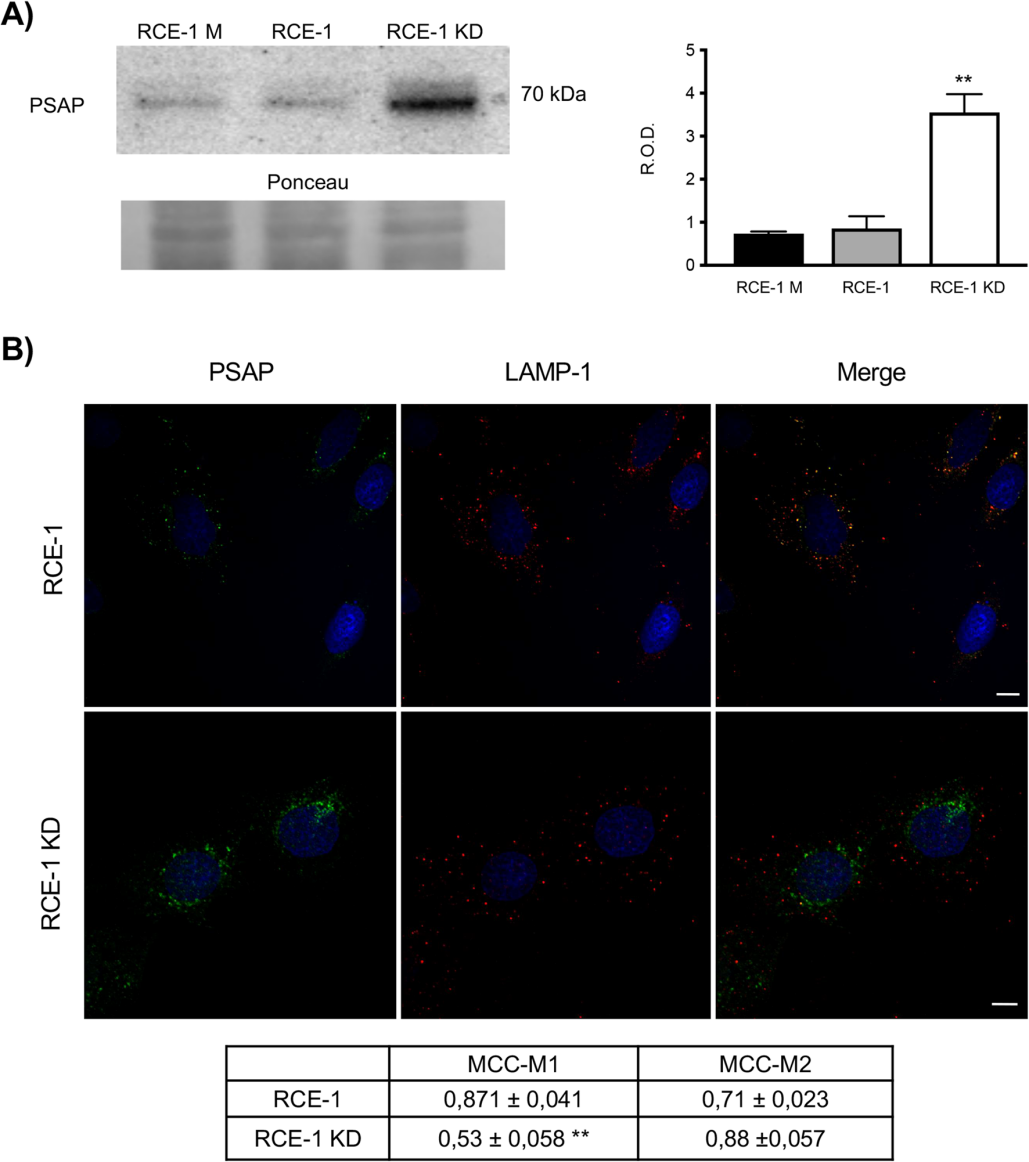
Effect of sortilin depletion on the expression and distribution of PSAP in RCE-1 cells. (**A**) Representative immunoblot of PSAP in RCE-1 M (mock-depleted), RCE-1 and RCE-1 KD cells and the corresponding quantification of bands. Bars represent the means of relative optical density (R.O.D.) ± SEM from three independent experiments. (**) significant difference from control and RCE-1 (p < 0.05). Ponceau S staining were used as loading control. The full-lenght immunoblot is presented in Supplementary Fig. 3. (**B**) Representative immunofluorescence staining of PSAP and LAMP-1 in RCE-1 and RCE-1 KD cells. Quantification of co-localization of PSAP and LAMP-1 (MCC-M1); LAMP-1 and PSAP (MCC-M2). Values are expressed as the means of Manders colocalization coefficients 1 and 2 (MCC-M1 and MCC-M2, respectively) ± SEM. (**) significant difference from RCE-1 (p < 0.05). Scale bars = 10 μm.

**Figure 3:**
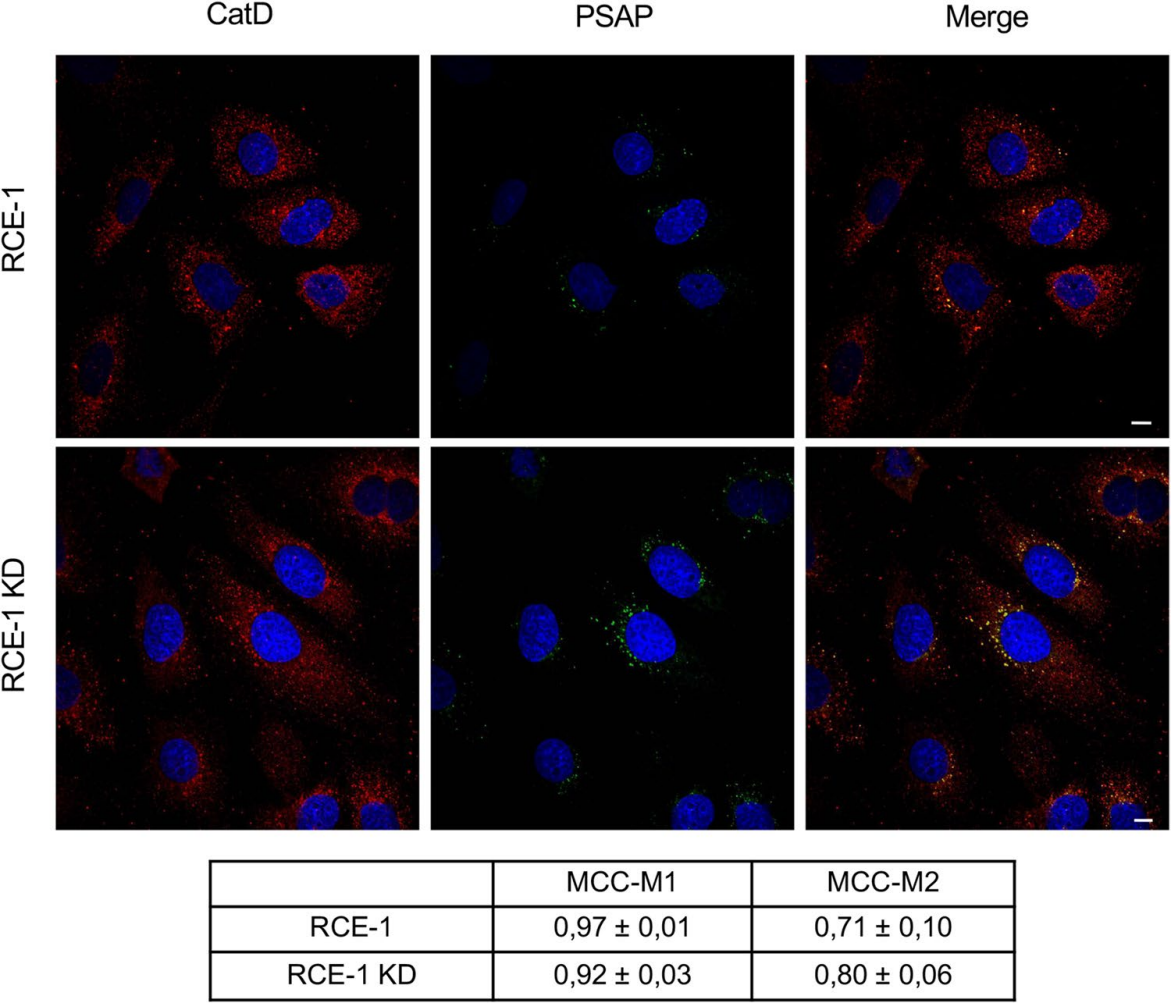
Distribution of PSAP and CatD in RCE-1 and RCE-1 KD cells. Representative immunofluorescence staining of PSAP and CatD in RCE-1 and RCE-1 KD cells. Quantification of co-localization of PSAP and CatD (MCC-M1); CatD and PSAP (MCC-M2). Values are expressed as the means of Manders colocalization coefficients 1 and 2 (MCC-M1 and MCC-M2, respectively) ± SEM. Scale bars = 10 μm.

Since the lysosomal protein PSAP has been described as a counterpart for the transport and processing of pro-CatD, we also studied the effect of sortilin knock-down on PSAP expression and distribution in epididymal cells. The basal expression of PSAP in epididymal cells is low, but it was significantly increased upon sortilin knock-down (Fig. 2A,B). Furthermore, PSAP redistribution from LAMP-1 positive compartments to a perinuclear vesicular location was also observed in the sortilin knock-down (Fig. 2B). As for the distribution of CatD, it was found that the enzyme mostly co-localizes with PSAP both in control cells (RCE-1) and in RCE-1 KD cells (Fig. 3).

### The sortilin knock-down (KD) induces changes in the CD-MPR

**Figure 4:**
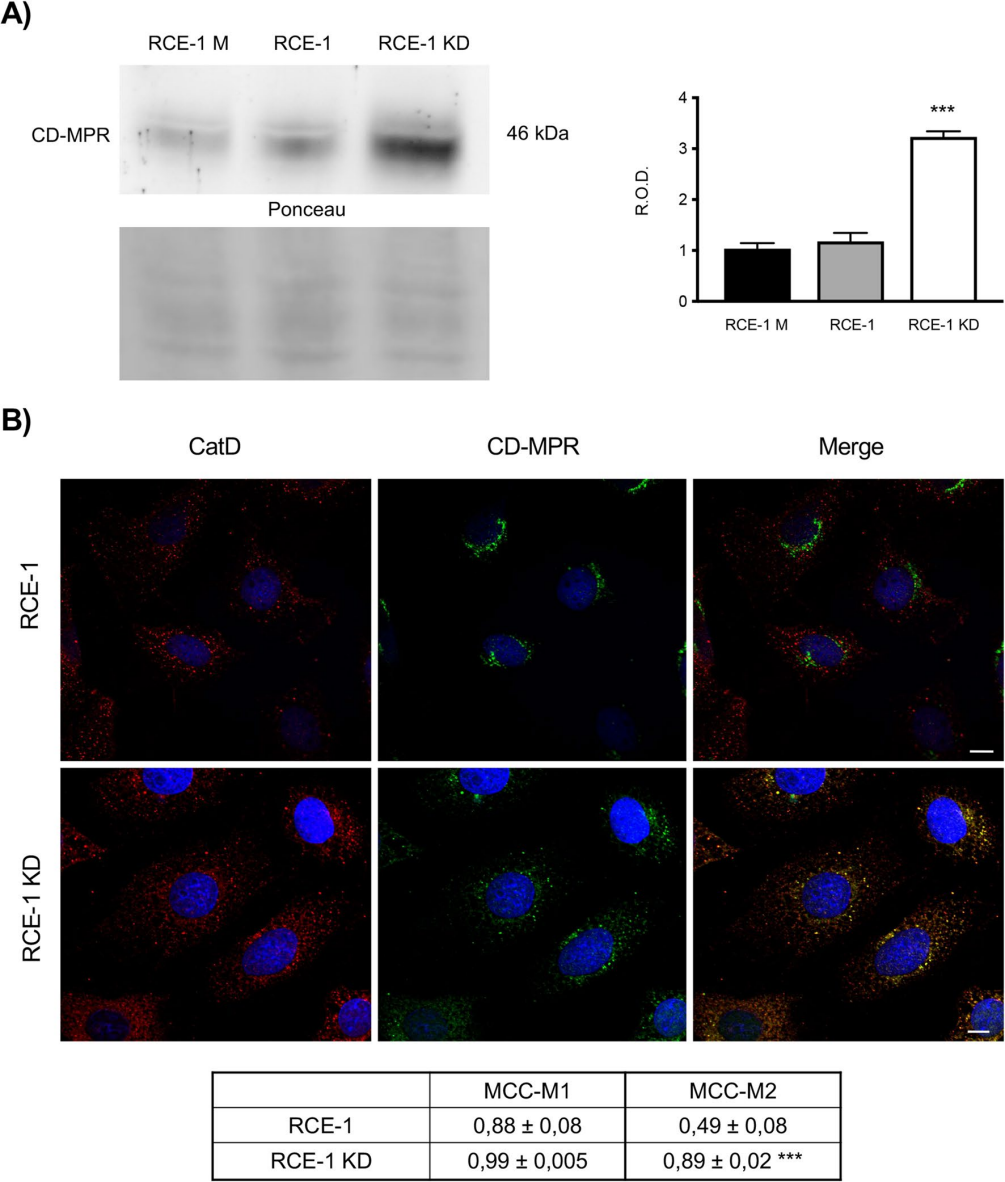
Expression and distribution of CD-MPR in RCE-1 and RCE-1 KD cells. (**A**) Representative immunoblot of CD-MPR in RCE-1 M (mock-depleted), RCE-1 and RCE-1 KD cells with their band intensity quantification. Bars represent the means of relative optical density (R.O.D.) ± SEM from three independent experiments. (***) significant difference from RCE-1 M and RCE-1 (p < 0.001). Ponceau S staining were used as loading control. The full-lenght immunoblot is presented in Supplementary Fig. 4. (**B**) Representative immunofluorescence staining of CD-MPR and CatD in RCE-1 and RCE-1 KD cells. Quantification of co-localization of CDMPR and CatD (MCC-M1); CatD and CD-MPR (MCC-M2). Values are expressed as the means of Manders colocalization coefficients 1 and 2 (MCC-M1 and MCC-M2, respectively) ± SEM. (***) significant difference from RCE-1 cells (p < 0.01). Scale bars = 10 μm.

In most cell types, CatD is commonly delivered to lysosomes via mannose-6-phosphate receptors (MPRs). Additionally, in some cellular models, the PSAP/sortilin-mediated pathway has been proposed to participate in CatD trafficking; however, it is not known whether both are alternative or redundant pathways for the enzyme. Therefore, we evaluated whether the sortilin silencing affects the state and distribution of the cation-dependent mannose-6-phosphate receptor (CD-MPR) in epididymal cells. By immunoblotting, we observed that the CD-MPR expression is significantly increased when the sortilin is knocked down in the RCE-1 cells (Fig. 4A). In addition, the CD-MPR was redistributed from a perinuclear location in the control cells to a cytoplasmic dispersed distribution in the RCE-1 KD cells (Fig. 4B). This phenomenon was accompanied by a significant increased co-localization of CD-MPR with CatD in the sortilin knocked down cells (Fig. 4B).

## Discussion

Mammalian epididymal cells have a highly developed lysosomal apparatus and maintain an adequate luminal environment for sperm maturation through the secretion of lysosomal proteins and other molecules^41–43^.

As in other cell types, the lysosome functionality depends on proper acidification and correct delivery of soluble lysosomal proteins to lysosomes^44,45^. In turn, the delivery of soluble lysosomal proteins to lysosomes is ensured primarily by mannose-6-phosphate receptors (MPRs). However, when the MPR-mediated pathway is non-functional, as it occurs in I-cell disease (Mucolipidosis type II), lysosomal proteins, such as CatD, can be transported by the sortilin receptor^30^. Sortilin was initially described as the neurotensin receptor-3, but its name was changed due to its involvement in the traffic of several proteins between intracellular compartments and/or to the cell surface.

PSAP is a lysosomal protein described as a natural ligand for sortilin and is selectively transported to lysosomes to be processed into saposins (A, B, C and D), which are required for the hydrolysis of glycosphingolipids^34,46^. It is also known that PSAP has a high affinity for CatD and that both may be transported to lysosomes via sortilin^30^. Moreover, it has been described that PSAP is involved in the processing of pro-CatD in acidic compartments^47^.

Although most mammalian cells release about 5–20% of soluble lysosomal proteins, part of which is recaptured via receptor-mediated endocytosis^48^, some cell types secrete higher amounts of lysosomal proteins into the extracellular compartment^9,10,41,49^. However, the mechanism of exocytosis as well as the function of these proteins in an extra lysosomal environment remain poorly understood.

One of the paradigms of lysosomal protein secretion occurs in the mammalian epididymis, whose epithelium synthesizes and releases abundant lysosomal proteins into the organ lumen^9,10,16,41,43^. Among them, CatD has been detected in the rat epididymal lumen, which is secreted as the 52 kDa precursor pro-CatD^10^ probably under androgen control. PSAP has also been found in epididymal fluid^50^ forming complexes with pro-CatD^16^. So far, the presence of these proteins in the epididymal fluid has been poorly explained and the mechanism of their release into the lumen is still unknown.

It could be possible that the mechanism of protein secretion is contingent to the intracellular transport. Thus, a correlation between the expression and distribution of receptors and lysosomal proteins could provide clues about the mode of secretion. In this regard, considerable levels of expression of both MPRs have been detected in rat^10,15^ and bovine epididymis^43^. In turn, sortilin has also been detected in rat epididymis (Supplementary Fig. 5) and efferent ducts^34^.

Although it has been reported that CatD is mainly transported via MPR in most cell types^51,52^, in this study we observed high co-localization levels between sortilin and CatD in the epididymal cell line RCE-1 (Fig. 1). This finding poses the question whether sortilin has a role in the trafficking of this enzyme along with PSAP in epididymal cells. To test this hypothesis, we have established the RCE-1 cell line, which has been depleted of sortilin (named RCE-1 KD cells) by an iRNA gene silencing method. After sortilin knockdown, a significant increase of PSAP expression was detected by immunoblotting. As observed in other sortilin-depleted cells^37^, PSAP redistributed from late endosomes and lysosomes to the perinuclear region in the epididymal RCE-1 KD cells, suggesting that PSAP accumulates in a compartment that prevents the processing to mature saposins. The redistribution of PSAP in RCE-1 KD cells was accompanied by CatD, with a concomitant decrease of this protease in LAMP-1 positive compartments.

Furthermore, our results showed that part of the perinuclearly accumulated PSAP co-localizes with CatD, suggesting that both proteins could be mostly complexed as reported in other cell types^29,47,53,54^, although, in this case, in a non-lysosomal compartment. Given that the processing of CatD occurs in lysosomes and that is impaired in RCE-1 KD cells, we propose that this enzyme mostly reaches the lysosomal compartment via a sortilin-PSAP mediated pathway. In turn, the intracellular accumulation of PSAP could be the result of a decreased activity of CatD, as it occurs in neural progenitor cells^55^.

It is worth mentioning that sortilin deficiency also induced a decrease of CatD levels (Fig. 1), which could be related to either an increased secretion of the enzyme because of its missorting or that sortilin somehow regulates the expression of CatD. Further studies (e.g. qPCR) are needed to confirm or rule out one these hypotheses. These results would suggest that sortilin participates, either directly or indirectly, in the transport of CatD and PSAP to lysosomes in principal cells of the epididymis.

Studies performed in another cell model, in which a dominant-negative sortilin construct and small interfering RNA (siRNA) was used, suggested that sortilin functions as an alternative sorting receptor to the MPR for CatD^30^. Interestingly, the sortilin depletion induced a significant increase in the CD-MPR expression in the epididymal cells, indicating that both receptors could regulate each other and that the observed increase in CD-MPR results from a compensatory mechanism for the accurate delivery of proteins to lysosomes. A similar reciprocal regulation has been demonstrated in other cell type^31^. In this regard, the sorting of sortilin is specifically upregulated in type II mucolipidosis to compensate the loss of MPR-dependent targeting of hydrolases. In addition to the increased expression of CD-MPR, we observed that this receptor is redistributed from a classic perinuclear location to a scattered and punctuated cytoplasmic location, accompanied by an increased co-localization with CatD. This suggests that CD-MPR is an alternative receptor to sortilin for the transport of CatD to lysosomes in epididymal cells.

The results obtained in this investigation would support the hypothesis that in RCE-1 cells CatD is transported to lysosomes by both the CD-MPR and/or the sortilin/PSAP pathways, and that CatD requires the presence of PSAP to be processed to the 48 kDa form^47^ in epididymal cells. In conclusion, these results have shown for the first time a compensatory response of CD-MPR to sortilin depletion and provided new insights into the mechanism of lysosomal protein transport in epididymal cells.

## Supplementary Information

Supplementary Figures.

## Data Availability

The full-length images of the original immunoblot membranes are available online in Supplementary Information. All data that support the findings of this study are available from the corresponding author on reasonable request.
