# Publisher Correction: Sortilin knock-down alters the expression and distribution of cathepsin D and prosaposin and up-regulates the cation-dependent mannose-6-phosphate receptor in rat epididymal cells

**DOI:** 10.1038/s41598-023-31255-x

**Published:** 2023-03-13

**Authors:** Andrea Carolina Aguilera, Natalia Leiva, Pablo Ariel Alvarez, Georgina Pulcini, Laura Lucía Pereyra, Carlos Ramón Morales, Miguel Ángel Sosa, Lorena Carvelli

**Affiliations:** 1 CONICET, Facultad de Ciencias Médicas, Universidad Nacional de Cuyo, M5500 Mendoza, Argentina; 2 Facultad de Ciencias Exactas y Naturales, Universidad Nacional de Cuyo, M5500 Mendoza, Argentina; 3 IHEM-CONICET, Facultad de Ciencias Médicas, Universidad Nacional de Cuyo, M5500 Mendoza, Argentina; 4 Faculty of Medicine, McGill University, Montreal, QC H3A0C7 Canada

Correction to: *Scientific Reports* https://doi.org/10.1038/s41598-023-29157-z, published online 01 March 2023

Figure 1 in the original version of this Article displayed a yellow border which was redundant and consequently removed.

The original Figure 1 and accompanying legend appear below.

**Figure 1:**
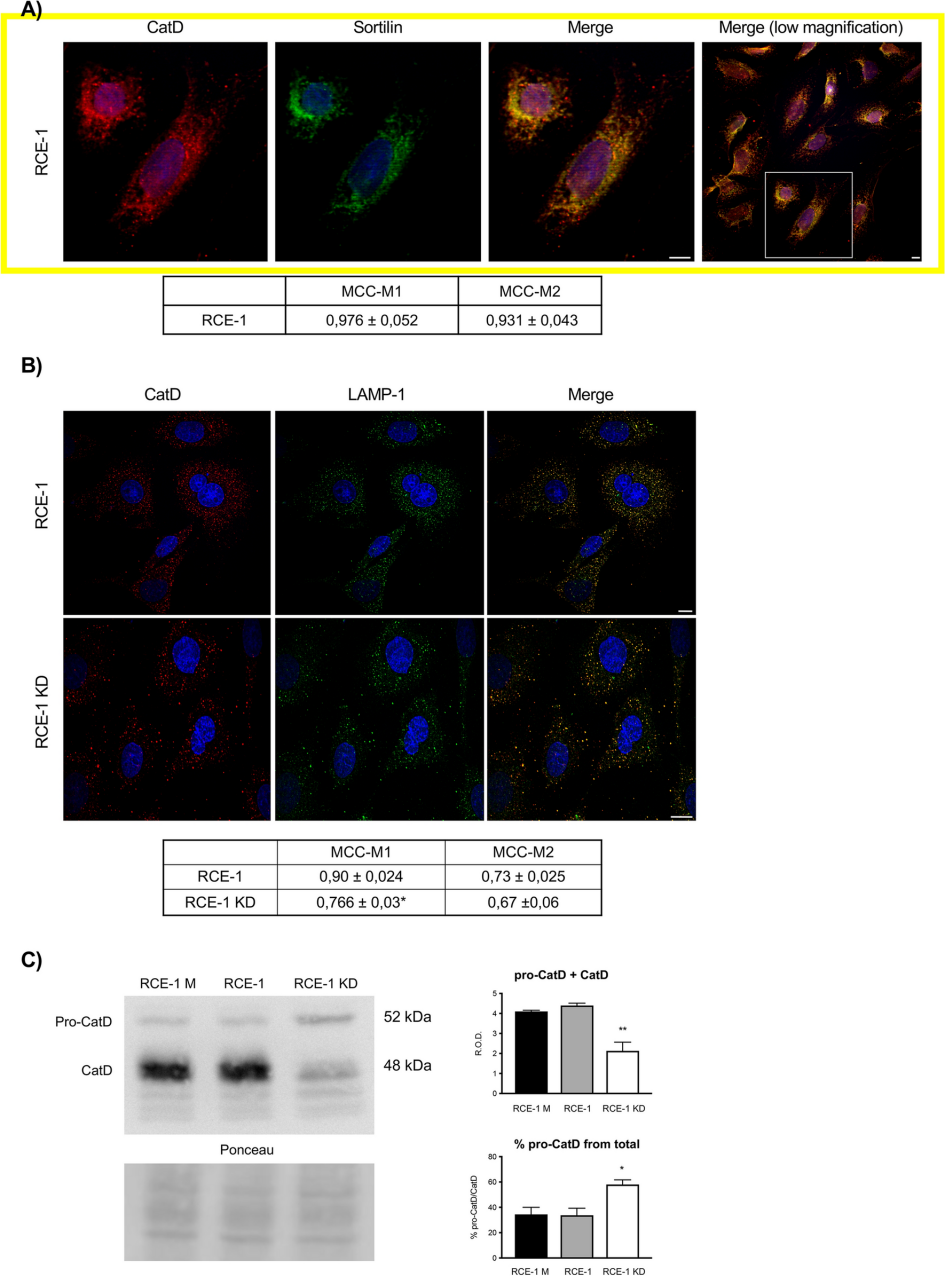
Effect of sortilin depletion on the distribution and processing of Cathepsin D in RCE-1 cells. (**A**) Immunofluorescence staining of sortilin and CatD in the RCE1 cells. Confocal representative images and quantification of co-localization of sortilin and CatD (MCC-M1); CatD and sortilin (MCC-M2). The shown cells were taken from the framed area of the lower magnification image (right merge). (**B**) Representative immunofluorescence staining of CatD and LAMP-1 in RCE1 and RCE-1 KD cells. Quantification of co-localization of LAMP-1 and CatD (MCC-M1); CatD and LAMP-1 (MCC-M2). Values are expressed as the means of Manders colocalization coefficients 1 and 2 (MCC-M1 and MCC-M2, respectively) ± SEM. (*) significant difference from RCE-1 (p < 0.01). Scale bars = 10 μm. (**C**) Representative immunoblot of CatD in RCE-1 M (mock-depleted), RCE-1 and RCE-1 KD cells. Bands intensities of pro-CatD (52 kDa) and CatD (48 kDa) were quantified separately. Bars represent the means of the total CatD (pro-CatD + CatD), as relative optical densities (R.O.D.) ± SEM (upper histogram) and the means of pro-CatD percentage in each sample ± SEM (lower histogram) from four independent experiments. (*) and (**) significant differences (p < 0.01 and p < 0.05, respectively). Ponceau S staining were used as loading control. The full-lenght immunoblot is presented in Supplementary Fig. 2.

The original Article has been corrected.

