# Supplementary material for: Sortilin knock-down alters the expression and distribution of cathepsin D and prosaposin and up-regulates the cation-dependent mannose-6-phosphate receptor in rat epididymal cells
Source: Sci Rep. 2023 Mar 1;13:3461. doi: 10.1038/s41598-023-29157-z (PMC9977780; doi:10.1038/s41598-023-29157-z)
Supplement: Supplementary file 1 — Supplementary Figures. [file 41598_2023_29157_MOESM1_ESM.pdf]

Supplementary Information

Sortilin knock-down alters the expression and distribution of cathepsin D and prosaposin and up-regulates the cation-dependent mannose-6-phosphate receptor in rat epididymal cells

Andrea Carolina Aguilera[1,2], Natalia Leiva[1,2], Pablo Ariel Alvarez[2], Georgina Pulcini[3], Laura Lucía Pereyra[3], Carlos Ramón Morales[4], Miguel Ángel Sosa[2,3], Lorena Carvelli[2,3*].

[1] CONICET, Facultad de Ciencias Médicas, Universidad Nacional de Cuyo, M5500, Mendoza, Argentina.

[2] Facultad de Ciencias Exactas y Naturales, Universidad Nacional de Cuyo, Mendoza, M5500, Argentina.

[3] IHEM-CONICET, Facultad de Ciencias Médicas, Universidad Nacional de Cuyo, M5500, Mendoza, Argentina.

[4] Faculty of Medicine, McGill University, Montreal, Quebec, H3A0C7, Canada.

* Corresponding author, fcarvelli@mendoza-conicet.gob.ar

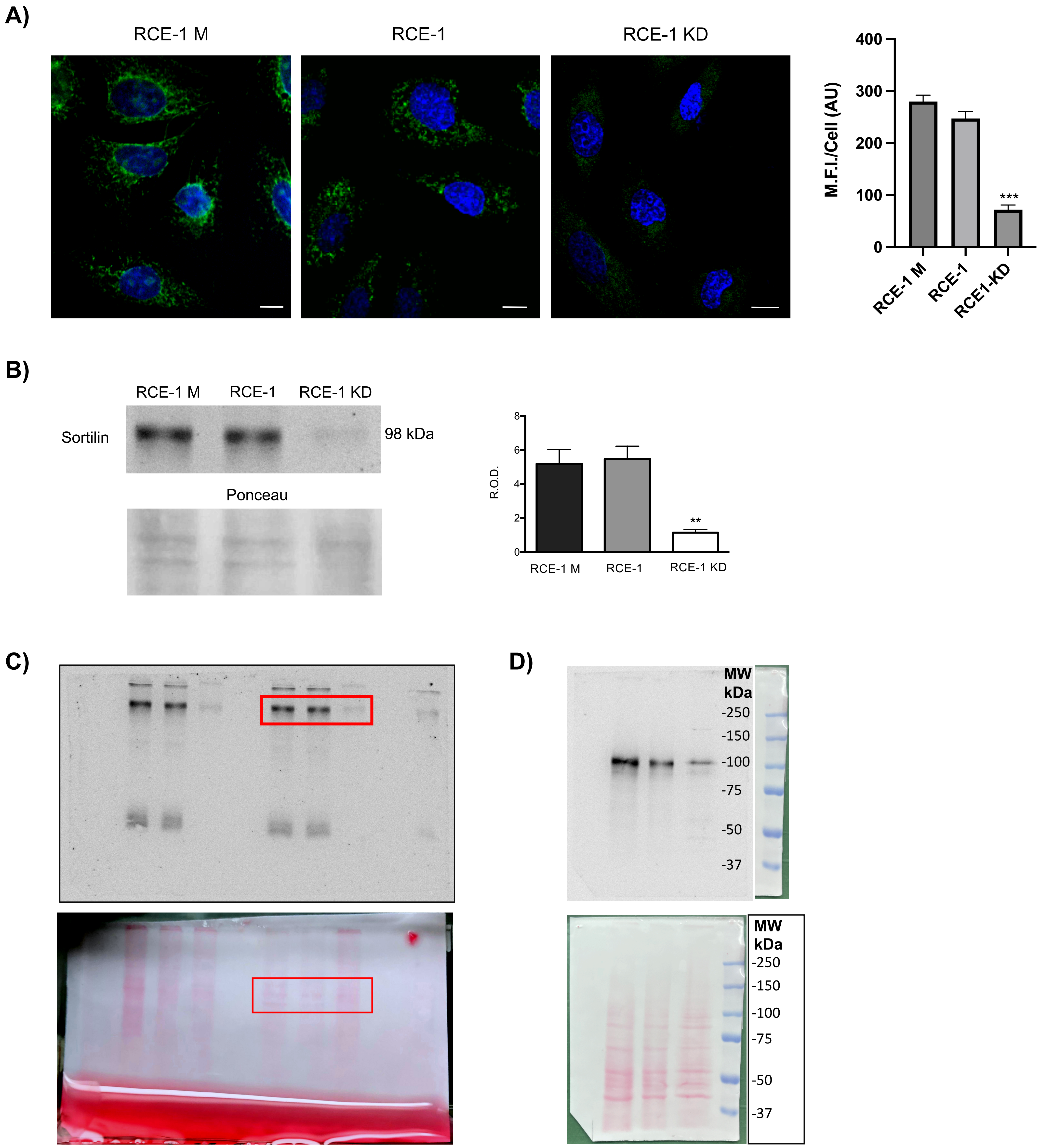

Supplementary Figure 1. Depletion of sortilin in RCE-1 epididymal cell line by pSilencer™ sortilin siRNA. A) Representative immunofluorescence staining of sortilin in RCE-1 M (mock-depleted), RCE-1 and sortilin knockdown RCE-1 cells (RCE-1 KD). Scale bar= 10 µm. Bars represent the means of green fluorescence intensity (M.F.I.) per cell ± SEM from three independent experiments. (***) significant difference from RCE-1 M and RCE-1 ($p < 0.001$). Data were analysed by one-way ANOVA followed by a Tukey´s multiple comparisons Test. AU: arbitrary units. **B)** Representative immunoblot of sortilin in RCE-1, RCE-1 M and RCE-1 KD cells with their band intensity quantification. Bars represent the means of relative optical density (R.O.D.) ± SEM from three independent experiments. (**) significant difference from RCE-1 M and RCE-1 ($p < 0.05$). Data were analysed by one-way ANOVA followed by a Tukey´s multiple comparisons Test. Ponceau S staining were used as loading control. **C)** Full-lenght immunoblot used in **B**. **D)** Another immunoblot of sortilin in RCE-1, RCE-1 M and RCE-1 KD cells. The corresponding Ponceau S staining shows the molecular weight markers. Molecular weight markers from ponceau image are shown in the corresponding immunoblot.

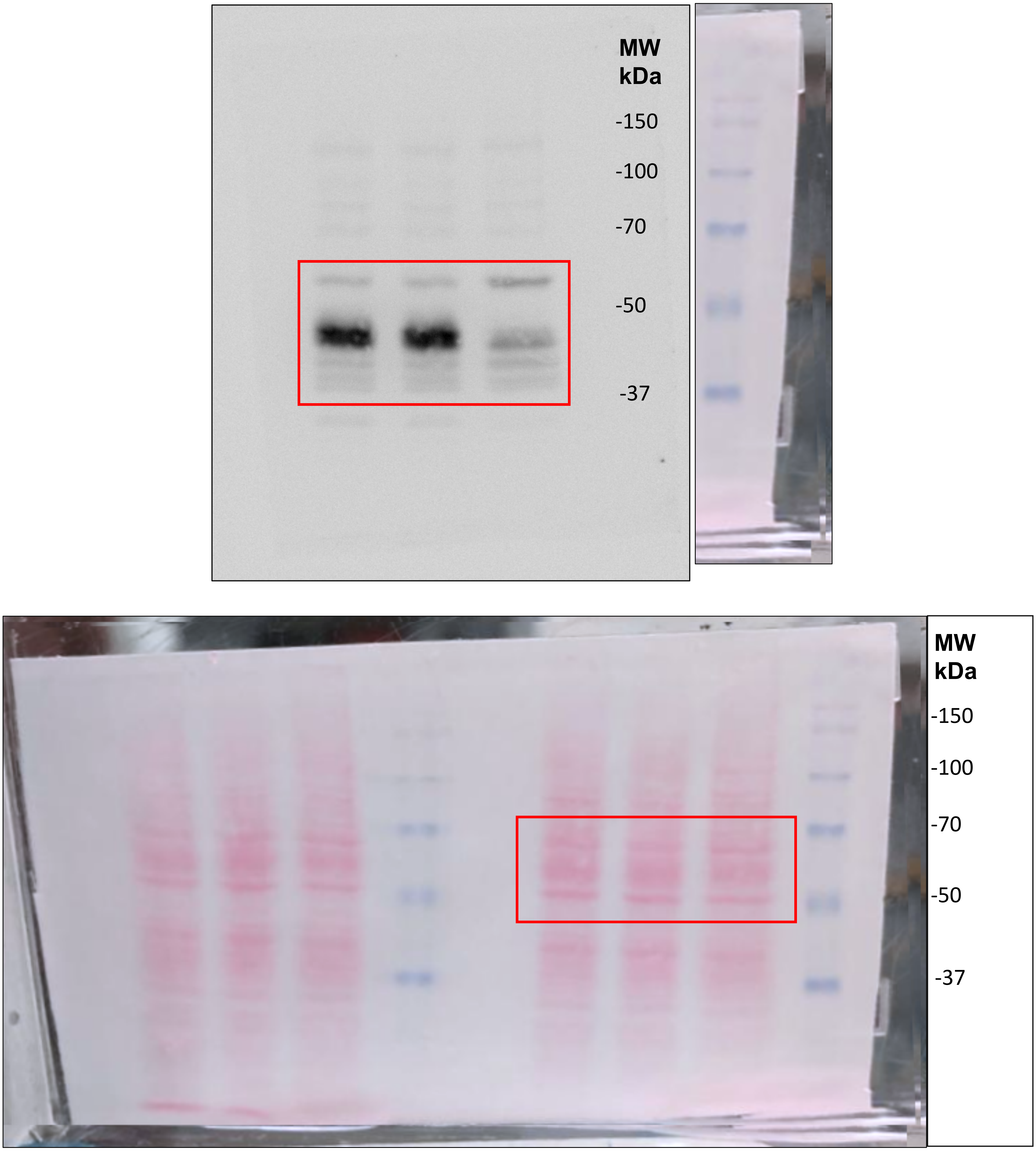

Supplementary Figure 2. Full-length immunoblot and Ponceau S staining which are shown cropped in Figure 1. Molecular weight markers from ponceau image are shown in the corresponding immunoblot.

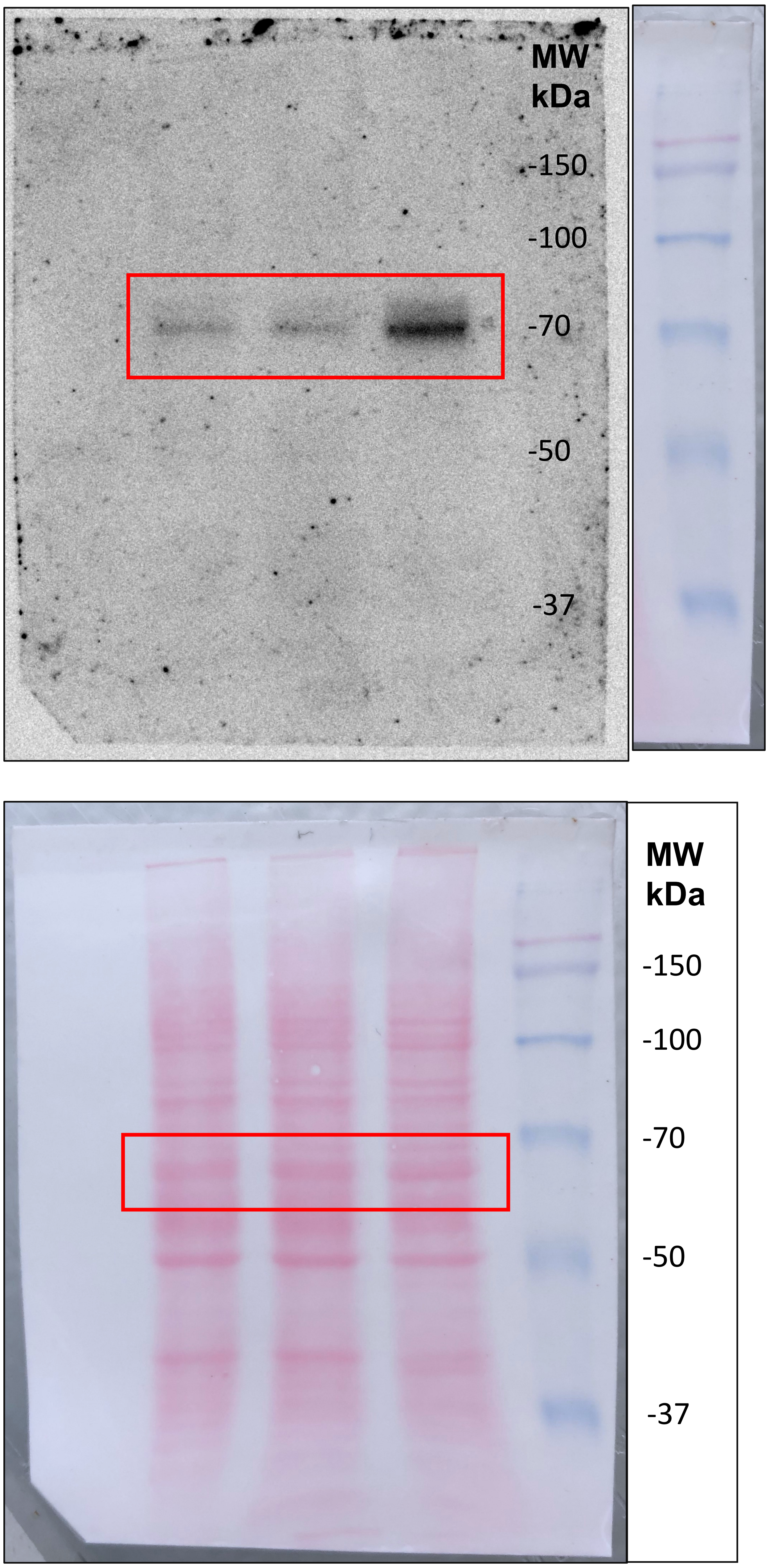

Supplementary Figure 3. Full-length immunoblot and Ponceau S staining which are shown cropped in Figure 2. Molecular weight markers from ponceau image are shown in the corresponding immunoblot.

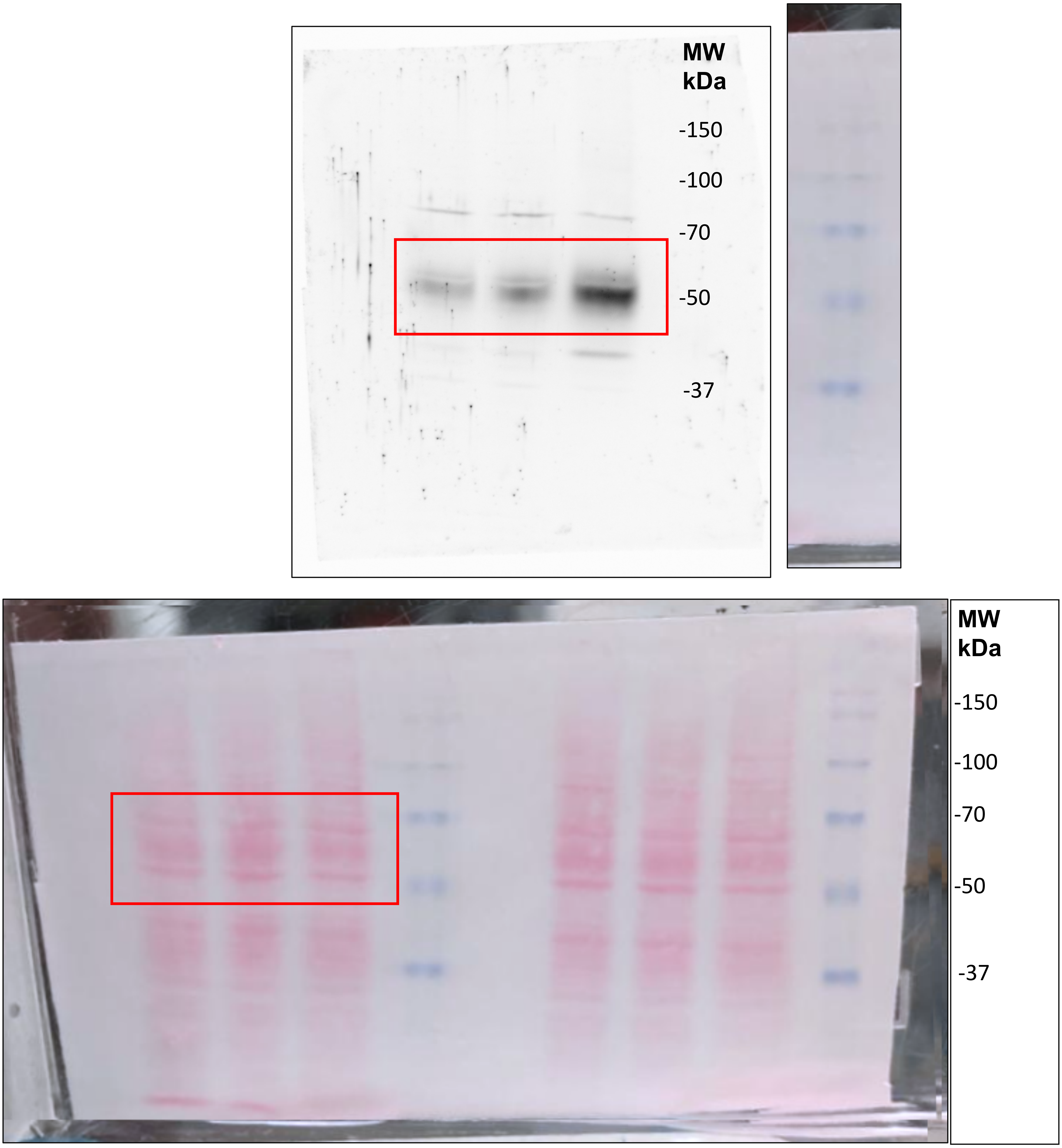

Supplementary Figure 4. Full-length immunoblot and Ponceau S staining which are shown cropped in Figure 4. Molecular weight markers from ponceau image are shown in the corresponding immunoblot.

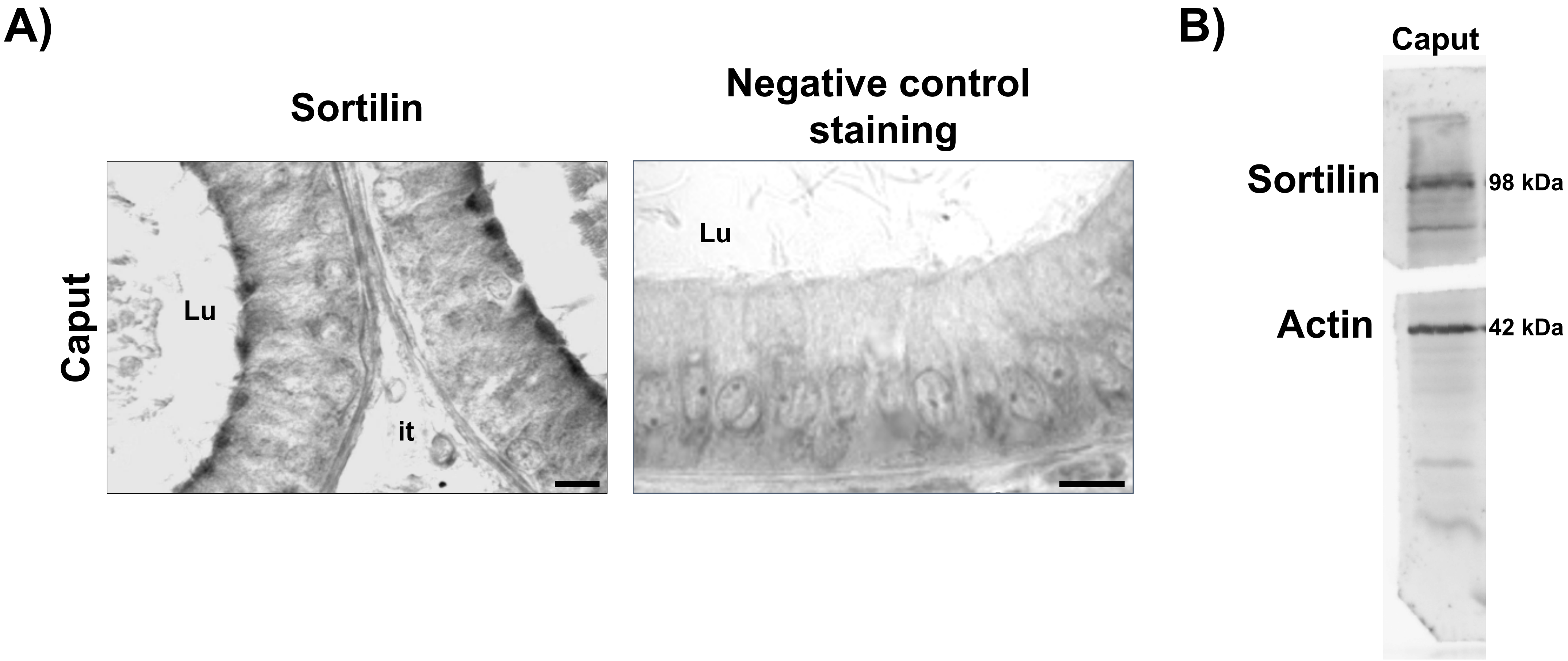

Supplementary Figure 5. Detection of sortilin in rat caput epididymis. Immunostaining **(A)** and immunoblot **(B)** of sortilin in caput epididymis from Sprague-Dawley rats (90 days old). Lu, lumen; it, interstitial space. Scale bars = 10 µm. Actin detection was used as loading control. The technical procedures were carried out according to Carvelli et. al., 2021 (Ref # 16).

The experimental procedures were reviewed and approved by the animal care and use committee of School of Medicine, Universidad Nacional de Cuyo (Institutional Committee for the Care and Use of Laboratory Animals, CICUAL; protocol reference number: 175/2019, http://fcm.uncuyo.edu.ar/paginas/index/cicual), in strict accordance with the recommendations in the Guide for the Care and Use of Laboratory Animals: Eighth Edition. Washington, DC: The National Academies Press. https://doi.org/10.17226/12910.
